# Biogenic Synthesis of Cu-Mn Bimetallic Nanoparticles Using Pumpkin Seeds Extract and Their Characterization and Anticancer Efficacy

**DOI:** 10.3390/nano13071201

**Published:** 2023-03-28

**Authors:** Nouf Omar Alafaleq, Torki A. Zughaibi, Nasimudeen R. Jabir, Azhar U. Khan, Mohd Shahnawaz Khan, Shams Tabrez

**Affiliations:** 1 Department of Biochemistry, College of Science, King Saud University, Riyadh, Saudi Arabia; 2 King Fahd Medical Research Center, King Abdulaziz University, Jeddah 21589, Saudi Arabia; 3 Department of Medical Laboratory Sciences, Faculty of Applied Medical Sciences, King Abdulaziz University, Jeddah 21589, Saudi Arabia; 4 Department of Biochemistry, Centre for Research and Development, PRIST University, Thanjavur 613403, India; 5 Department of Chemistry, School of Life and Basic Sciences, Siilas Campus, Jaipur National University, Jaipur 302017, India; 6 Protein Research Chair, Department of Biochemistry, College of Science, King Saud University, Riyadh 11451, Saudi Arabia

**Keywords:** biosynthesis, bimetallic nanoparticles, cytotoxicity, pumpkin, spectral analysis

## Abstract

Background: Cancer is a chronic, heterogeneous illness that progresses through a spectrum of devastating clinical manifestations and remains the 2nd leading contributor to global mortality. Current cancer therapeutics display various drawbacks that result in inefficient management. The present study is intended to evaluate the anticancer potential of Cu-Mn bimetallic NPs (CMBNPs) synthesized from pumpkin seed extract against colon adenocarcinoma cancer cell line (HT-29). Methods: The CMBNPs were biosynthesized by continuously stirring an aqueous solution of pumpkin seed extract with CuSO4 and manganese (II) acetate tetrahydrate until a dark green solution was obtained. The characteristic features of biogenic CMBNPs were assessed by UV-visible spectrophotometry (UV-vis), X-ray powder diffraction (XRD), energy-dispersive X-ray (EDX), scanning electron microscopy (SEM), and transmission electron microscopy (TEM). A battery of biological assays, viz. neutral red uptake (NRU) assay, in vitro scratch assay, and comet assay, were performed for anticancer efficacy evaluation. Results: The formation of spherical monodispersed bimetallic nanoparticles with an average size of 50 nm was recorded using TEM. We observed dose-dependent cytotoxicity of CMBNPs in the HT-29 cell line with an IC_50_ dose of 115.2 µg/mL. On the other hand, CMBNPs did not show significant cytotoxicity against normal cell lines (Vero cells). Furthermore, the treatment of CMBNPs inhibited the migration of cancer cells and caused DNA damage with a significant increase in comet tail length. Conclusions: The results showed substantial anticancer efficacy of CMBNPs against the studied cancer cell line. However, it is advocated that the current work be expanded to different in vitro cancer models so that an in vivo validation could be carried out in the most appropriate cancer model.

## 1. Introduction

Despite a significant understanding of the molecular pathways involved, cancer remains the 2nd leading cause of death, accounting for one in every six global mortalities [1,2]. Cancer is well known as a chronic, heterogeneous illness that arises at the genetic, phenotypic, and pathological levels and progresses through a spectrum of clinical manifestations [3]. Currently, multiple cancer therapeutics experience setbacks of drug resistance, are unresponsive, and have severe side effects [4]. In this scenario, cancer treatment approaches must devise new, novel, and innovative therapeutic strategies to overcome the above-mentioned limitations. Several plant-originated small molecules have shown significant anticancer effects, overcoming biocompatibility, multidrug resistance, and perceived toxicity [5,6]. In addition, most anticancer therapeutics have failed clinical trials due to non-specific interactions and adverse side effects at pharmacologically relevant concentrations [7].

Nanotechnology offers an innovative, robust, and flexible drug delivery platform to overcome the limitations of conventional chemotherapy and can control 3D molecular structures to build materials/devices with precision [8,9]. The multiple properties of nanoparticles (NPs), including smaller size, high surface-to-volume ratio, unique fluorescent features, increased permeability, and remarkable biocompatibility, offer several advantages in cancer treatment [10]. Among the nanoparticles, metallic NPs have great potential to be utilized as pharmacological agents and exhibit a superior performance compared to other NPs [11]. Owing to their unique optical, electrical, magnetic, and catalytic capabilities, which are typically different from those of their monometallic counterparts, bimetallic NPs have drawn much attention in the last decade. Bimetallic nanoparticles are composed of two different metal elements and have gained importance because of their better potential compared to monometallic NPs, usually possessing superior activity, selectivity, and stability [12]. Due to the synergistic characteristics between the two separate metal components, bimetallic NPs typically display more intriguing attributes than the corresponding monometallic NPs [13]. On the other hand, the biosynthesis of bimetallic nanoparticles is a clean, green, inexpensive, safe, and eco-friendly method compared to chemical and physical methods [14,15].

Recently, some bimetallic NPs, including, Ag-Au, Ag-Cu, Zn-Cu, and Zn-Ag have been investigated for their anticancer effect and showed better efficacy compared to their monometallic NPs [16,17,18,19]. The current study biosynthesized Cu-Mn bimetallic NPs (CMBNPs) from pumpkin seed extract and evaluated their anticancer effects against the colon adenocarcinoma cancer cell line (HT-29).

## 2. Materials and Methods

### 2.1. Materials

Pumpkin seeds were collected from Jaipur market, Rajasthan, India. Copper acetate and manganese (II) acetate tetrahydrate were obtained from Merck and Sigma-Aldrich, India, respectively. The study’s other reagents were all purchased locally.

### 2.2. Preparation of Seed Extract

The pumpkin seeds were cleansed with tap water to eliminate dirt and other unwanted objects, rinsed with double-distilled water and then allowed to air-dry at room temperature. A total of 10 gm of finely grinded pumpkin seeds were added to 150 mL of double-distilled water in a 250 mL round bottom flask, and the mixture was refluxed for 45 min. The extract was cooled to room temperature for downstream processing and filtered through Whatman filter paper no. 1.

### 2.3. Biogenic Synthesis of CMBNPs

Double-distilled water was used to dissolve 5 mM CuSO_4_ and 5 mM manganese (II) acetate tetrahydrate (1:1 ratio; 80 mg/mL). A light blue colloid was formed at room temperature after constant stirring of the mixture for 5 min. Adding pumpkin seed extract aqueous solution and heating it to 90 °C produced a dark green solution. The reaction mixture was then centrifuged at 7000 rpm for 10 min to remove unreactive plant components and metal precursors. Following centrifugation, the reaction mixture was cleaned with ethanol and double-distilled water before being air-dried at 50 °C and stored in a cold environment.

### 2.4. Characterization of CMBNPs

The biogenic synthesis of CMBNPs was characterized by the variety of spectral methods available, including Fourier transform infrared spectroscopy (FTIR), X-ray diffraction (XRD), energy-dispersive X-ray spectroscopy, scanning electron microscopy (SEM), and transmission electron microscopy (TEM).

### 2.5. Cell Culture Maintenance

The National Centre for Cell Sciences (NCCS), Pune, India, provided colon adenocarcinoma cell line (HT-29) and Vero cells (normal cells), which were then cultivated in a complete growth medium (DMEM) with 10% fetal bovine serum (FBS), penicillin (100 U/mL), and streptomycin (100 µg/mL). The cells were kept at 37 °C in a humidified incubator with 5% CO_2_.

### 2.6. Neutral Red (NR) Uptake Assay

This assay determines the rate of cell growth inhibition based on the principle that only living cells can take up the red dye and absorb it in lysosomes, whereas dead or damaged cells cannot [20]. After incubating the cells with various concentrations of CMBNPs (0–1000 µg/mL), 10 µL of neutral red (40 µg/mL in PBS) was added to each well and incubated for 1 h. Subsequently, the medium was decanted, and NR was dissolved with a destained solution. A microplate reader measured the absorbance at 550/660 nm (Biotek, Charlotte, VT, USA). The IC_50_ values were determined using a calibration curve, which indicates the sample concentrations required to inhibit 50% of cell growth.

### 2.7. In Vitro Scratch Assay

The scratch assay was performed to examine the impact of CMBNPs on cell–cell interactions and cell migration [21,22]. The HT-29 cells were seeded in a 6-well plate for 24 h at 37 °C with 5% CO_2_ in complete media. After 24 h, cells were scratched with a sterile 200 µL micropipette tip, treated with different concentrations of CMBNPs and photographed at specific time intervals from 0–72 h. The area of the gap was analyzed using Image J software.

### 2.8. Comet Assay

115 μg/mL of CMBNPs was applied to the seeded HT-29 cells for 24 h. Following a slightly modified version of the methodology of Tice et al. [23] and Singh et al. [24], only cell suspensions with more than 90% viabilities were utilized to determine DNA damage using the alkaline variant of the comet test. After being re-suspended, the treated cells were mixed with low-melting-point agarose at 37 °C and put on microscope slides that had already been coated with normal-melting agarose. After that, the slides were covered by a cover slip and kept at 4 °C for 15 min. The slides were then uncovered and incubated in a lysis buffer (2.5 M NaCl, 100 mM EDTA, 10 mM trizma base, 1% triton X-100, 10% DMSO, pH 10) at 4 °C for 1 h in the absence of light. The slides were then put into an electrophoresis chamber at 4 °C and treated with an alkaline electrophoresis buffer (300 mM NaOH, 1 mM EDTA, pH > 13) in the dark for 40 min before being put through electrophoresis (25 V and 300 mA) in the dark at 4 °C for 10 min. Following electrophoresis, the slides were dried, placed in tight containers, and washed for 15 min in a buffer (0.4% Tris, pH 7.5). A measure of DNA damage was calculated using the mean tail intensity.

## 3. Results and Discussion

### 3.1. UV-Vis Spectroscopy

**Figure 1:**
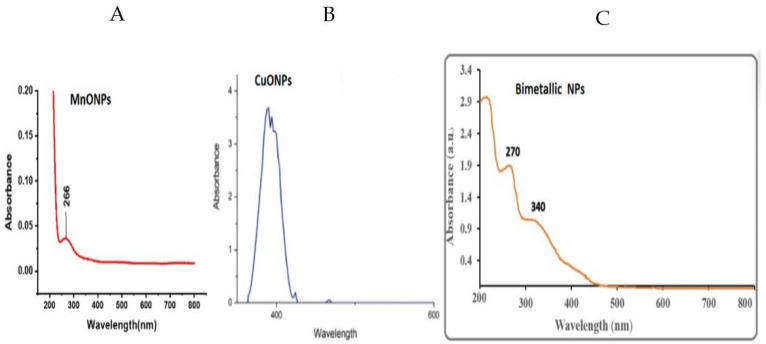
UV-vis spectra of MnONPs (**A**), CuONPs (**B**), and Cu-Mn bimetallic nanoparticles (**C**) biosynthesized from pumpkin seed extract.

UV-vis spectroscopy is a useful technique used to confirm the formation and stability of metal nanoparticles in aqueous solutions. The presence of the surface plasmon resonance (SPR) bands of monometallic NPs (CuNPs and MnONPs) in the UV-vis of the bimetallic NPs demonstrated that the bimetallic NPs were successfully produced using the pumpkin seed extract. The SPR band maxima of NPs are 336 nm and 266 nm for monometallic CuONPs and MnONPs, respectively (Figure 1A,B). Figure 1C exhibited the UV-vis spectra of CMBNP formation utilizing constant concentrations of copper acetate, manganese acetate tetrahydrate, and pumpkin seed extract. The gradual color change from light green to dark green during the reaction and differing amounts of pumpkin seed extract resulted in surface plasmon resonance (SPR).

The SPR bands of bimetallic NPs differ from the absorption bands of Cu and Mn monometallic NPs. The bimetallic Cu-Mn NPs have SPR band maxima of 270 and 340 nm, showing a slight redshift of Mn and some degree of blueshift of Cu in a bimetallic nanoparticle (Figure 1C).

### 3.2. FTIR Spectroscopy

**Figure 2:**
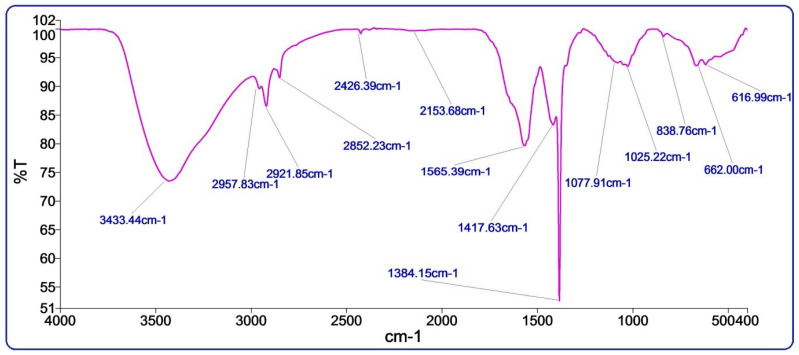
FTIR spectra of Cu-Mn bimetallic nanoparticles.

The FTIR spectra were recorded to identify bioactive functional groups in the pumpkin seeds extract (Figure 2). The peaks at 3363, 2957, 2921 cm^−1^ showed strong stretching vibration frequency of hydroxyl and aromatic –C=C– groups [25]. The −O-H band stretching at 3363 cm^−1^ describes the interaction of organic molecules with Cu-Mn bimetallic nanoparticles. On the other hand, the peak at 1416 cm^−1^ indicates a C-N group. The absorption peak at 1384 cm^−1^ assigns the adsorbed water of Mn nanoparticles [26]. The stretching bands at 1565, 1075, and 1021 cm^−1^ showed the absorption of primary aliphatic amines and other secondary metabolites, the presence of a hydroxyl group, and possible metals reduction, indicating the presence of bioactive compounds such as phenolic, fatty acids, and tocopherols [25]. The two consequence peaks at 838 and 582 cm^−1^ characterize stretching bonds O-Mn-O, indicating the appearance Cu-Mn bimetallic nanoparticles in the sample [26,27].

### 3.3. X-ray Diffraction (XRD) Pattern of Cu-Mn Bimetallic Nanoparticles

**Figure 3:**
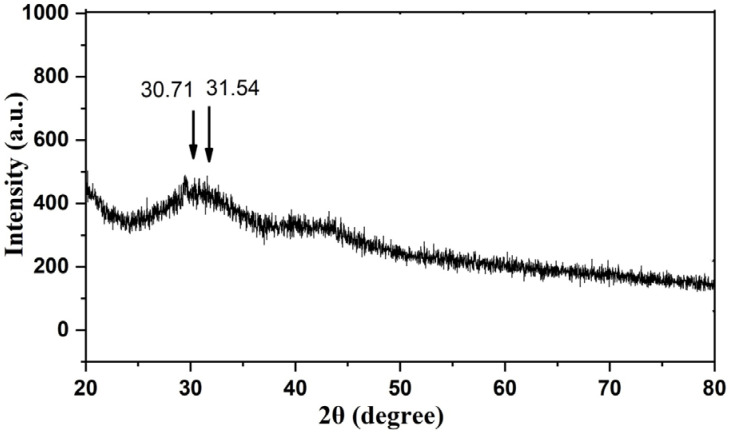
XRD pattern of Cu-Mn bimetallic nanoparticles.

XRD pattern was used to describe the crystalline structures of Cu-Mn. The signals of Cu and Mn in bimetallic nanoparticles showed separate diffraction peaks at 30.71 and 31.54 in the XRD at the 2θ position, corresponding to the Brags peaks of (200) and (011), respectively (Figure 3). These are attributed to a tetragonal and an orthorhombic phase. The values displayed on the XRD pattern were compared to the JCPDS cards 83–1665 and 89–1397 and were found to be nearly identical [28]. The results showed typical peaks with lowered intensity and significant widening. The lower intensities are due to the obstructive effect of the amorphous particles.

### 3.4. Energy-Dispersive X-ray (EDX) Analysis of Cu-Mn Bimetallic Nanoparticles

**Figure 4:**
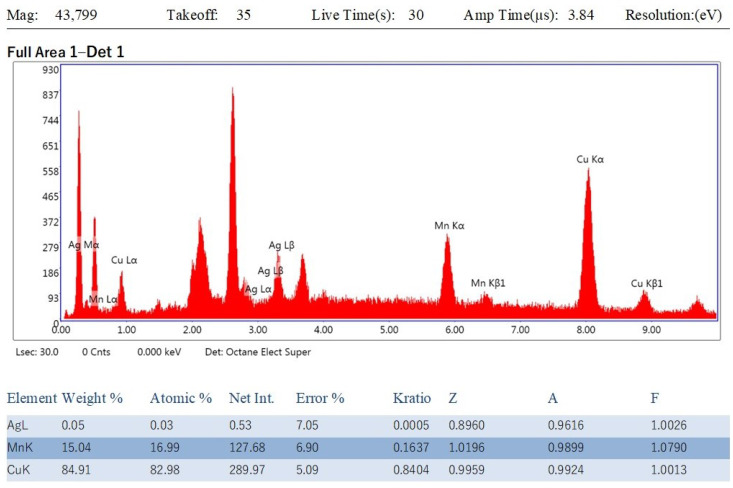
EDX spectra and elemental composition of biosynthesized Cu-Mn bimetallic nanoparticles.

The EDX spectrum described elemental composition with the weight and atomic percentage of Cu and Mn in the biosynthesized nanoparticles (Figure 4). The EDX elemental composition exhibited 84.91% copper and 15.04% manganese in nanoparticles, supporting the fabrication of Cu-Mn nanoparticles [25,27].

### 3.5. Scanning Electron Microscopy (SEM)

**Figure 5:**
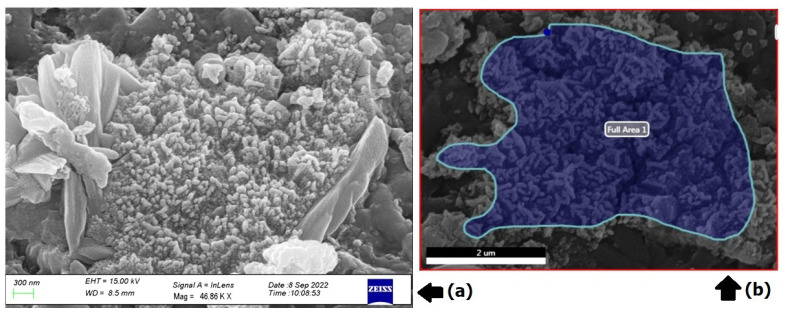
SEM images of Cu-Mn bimetallic nanoparticles at lower (**a**) and higher (**b**) magnification.

Cu-Mn bimetallic nanoparticles’ surface morphology was examined using scanning electron microscopy. Figure 5a,b show representative SEM pictures at various magnifications. At the lower magnification, it can be realized that even though the particles are well distributed, the boundaries between each particle are not clearly visible, while at the higher magnification, the surface morphology of granules and mono-dispersive clusters is visible in the form of assemblies and in the boundaries between each particle (Figure 5b). The cluster of particles has formed because of the attraction forces that bind them together. The average particle size, as seen in the SEM image, was 300 nm.

### 3.6. TEM Analysis

**Figure 6:**
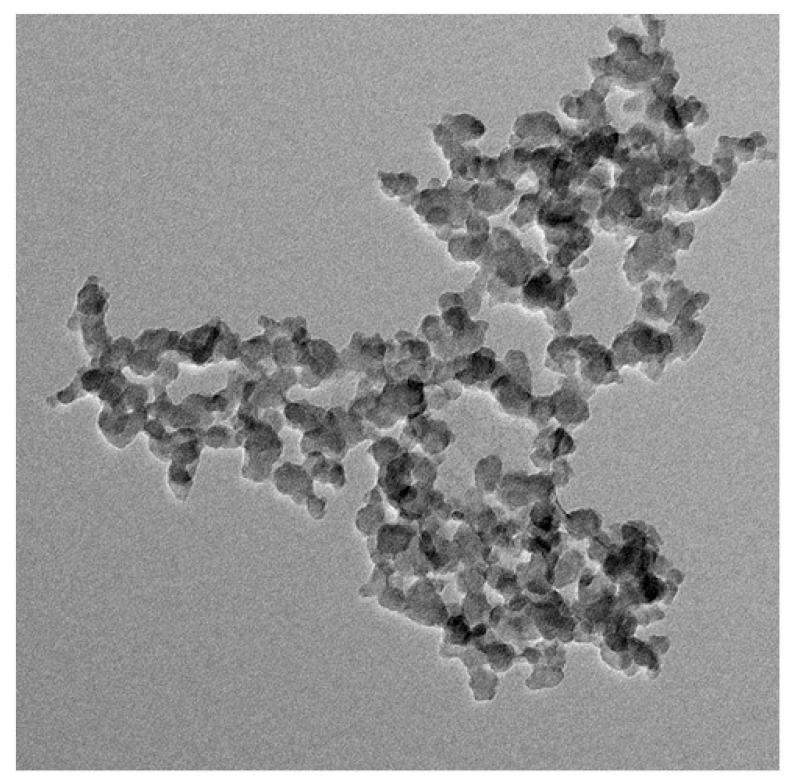
TEM analysis showing different sizes of Cu-Mn bimetallic nanoparticles with an average particle size of 50 nm. Scale bar indicates 100 nm.

The size and surface morphology of biosynthesized Cu-Mn bimetallic nanoparticles were characterized by TEM analysis (Figure 6). TEM is a handy technique that gives information concerning particle size distribution, mean particle size, and shape of nanoparticles. The biosynthesized Cu-Mn bimetallic nanoparticles were found to be spherical, and their size was found to be in the range of 50 nm. The chemical characterization by SEM, EDX, and TEM demonstrated the successful fabrication of Cu-Mn bimetallic nanoparticles from pumpkin seed extract [25,27].

### 3.7. Cytotoxicity Analysis

**Figure 7:**
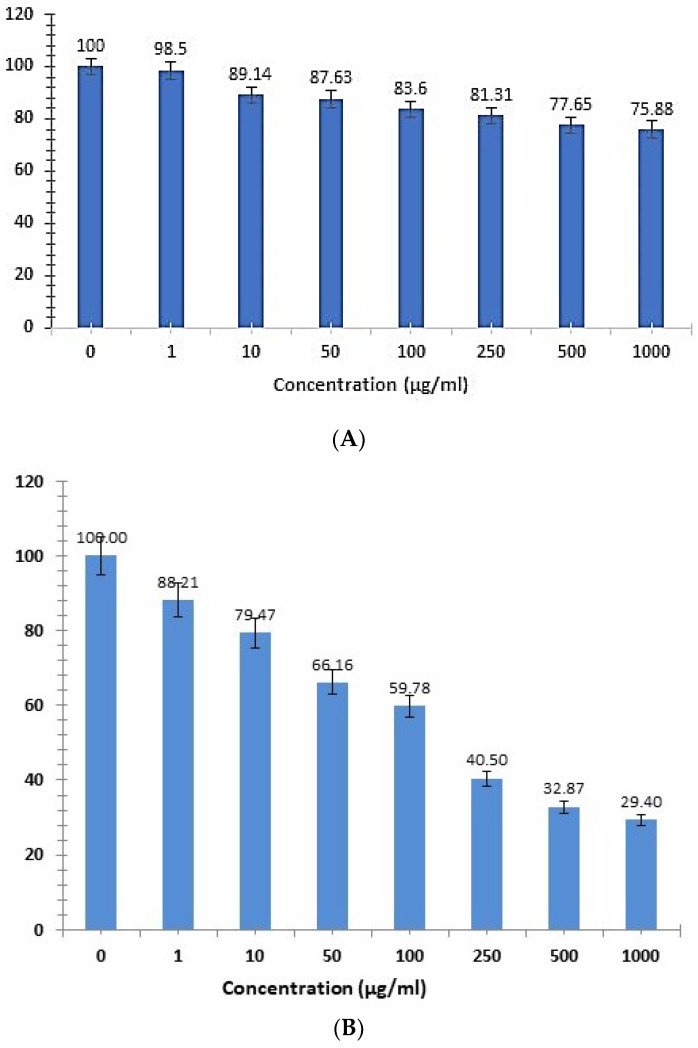

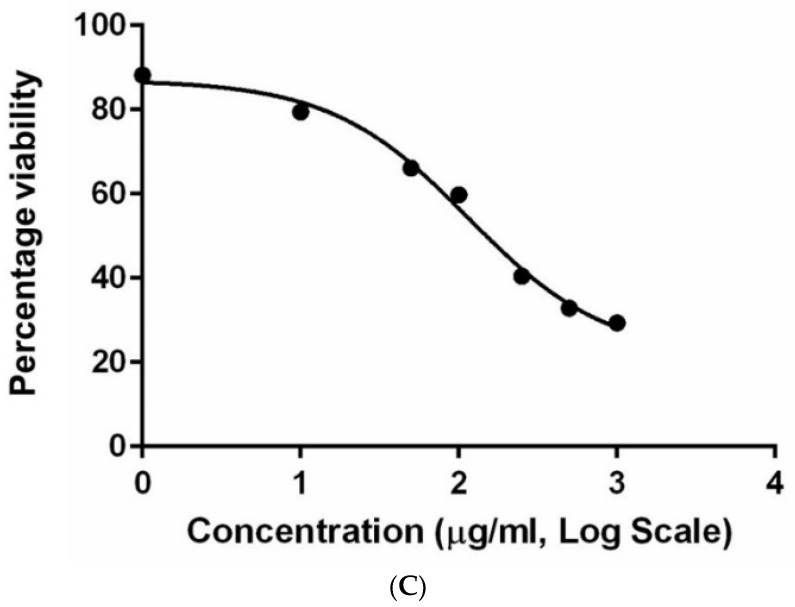
NRU assay depicting insignifcant increase in cytotoxicity in Vero cells (Normal cell line, (**A**)) and dose-dependent increased cytotoxicity in HT-29 cell lines (**B**). The data are presented as a percentage of viable cells compared to the untreated control (DMSO) cells, and estimated IC50 value was 115.2 µg/mL (**C**).

The cytotoxicity of green synthesized CMBNPs was determined by using a NRU colorimetric assay. This assay is based on the capacity of live cells to bind and integrate neutral red into lysosomes [29]. The cytotoxicity was measured as a concentration-dependent reduction in the uptake of neutral red in response to CMBNP exposure and serves as a sensitive indicator of both cell integrity and growth inhibition, determining IC_50_ (50% inhibiting concentration). We observed a decline in the cell viability of HT-29 cells with an increasing concentration of CMBNPs compared to untreated control (DMSO) cells (Figure 7). All treated HT-29 cells were found to be inhibited by different concentrations of CMBNPs (1–1000 µg/mL). In contrast, the viable cell counts gradually decreased with increasing CMBNPs concentration and went below 29.4% at the highest tested concentration (1000 µg/mL). The dose-dependent curve was used to calculate the IC_50_ value of CMBNPs as 115.2 µg/mL. On the other hand, CMBNPs did not show significant cytotoxicity against normal cell lines (Vero), even at a 1000 µg/mL concentration (Figure 7A). Ahmad et al. (2022) recently reported the better cytotoxicity potential of manganese-doped copper NPs which had been synthesized from *vinca rosea* extract [27]. Similarly, Zadeh et al. (2022) reported the dose-dependent cytotoxic effects of biosynthesized copper/zinc bimetallic nanostructures on MCF-7 cancer cell lines [30]. The above studies clearly highlight the advantage of bimetallic NPs compared to their monometallic counterpart.

### 3.8. Scratch Assay

**Figure 8:**
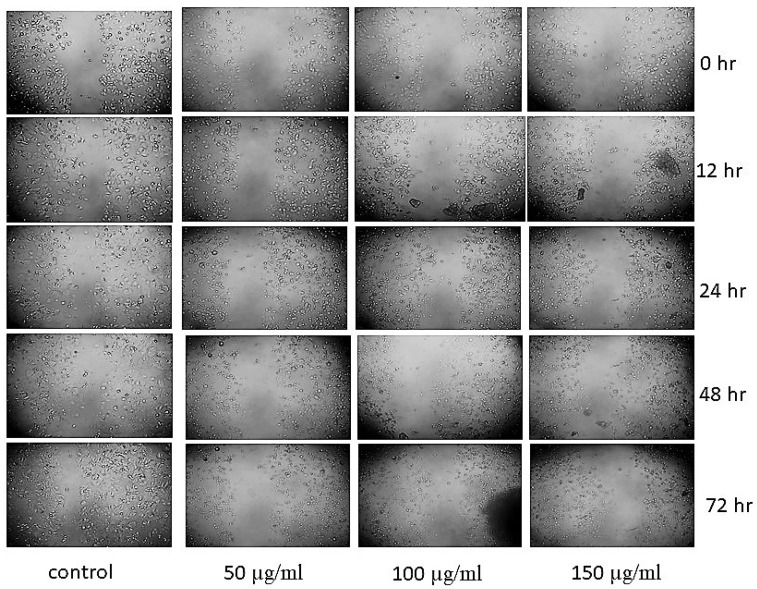
Scratch assay showing the inhibition of cellular migration of HT-29 cells after the treatment with CMBNPs.

Cell migration and invasion are essential for the spread of primary tumors to metastases, which play a crucial role in the poor prognosis of cancer [31]. This study used HT-29 cells derived from a more advanced adenocarcinoma of Dukes stage C, an optimal model to use to study metastatic progression in colorectal cancer [32]. Considering the interesting in vitro antiproliferative activity of CMBNPs, we performed a scratch wound healing assay to gauge its effectiveness as an anti-metastatic agent. In this assay, the effect of migration was monitored over time through a series of images captured immediately after scratch and treatment after 0, 12, 24, 48, and 72 h. The treatment of different concentrations of CMBNPs showed a considerable reduction in cell migration for wound closure compared to the control (Figure 8). The anti-migratory effects were more evident/noticeable at the highest tested concentrations of CMBNPs and at longer treatment durations. Several studies also reported different nanoformulations potential abilities to inhibit wound closure and cellular migration in various cancer cell lines [22,33,34].

### 3.9. CMBNP-Induced DNA Damage in HT-29 Cells

**Figure 9:**
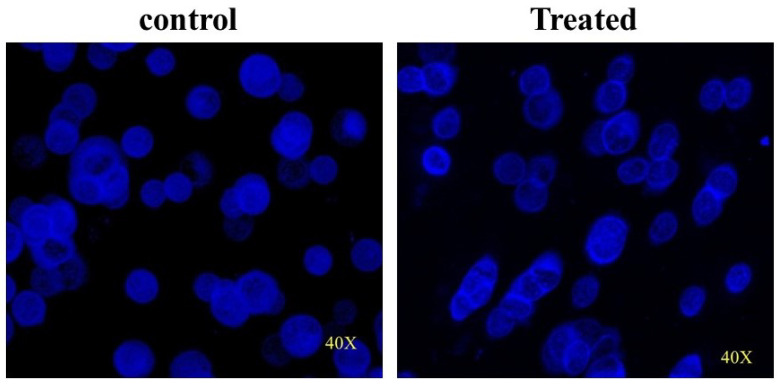
Comet image depicting difference in treated and control HT-29 cells (40×).

The comet assay is widely used to detect DNA damage and make repairs in response to genotoxic stress. It is a reliable method to use to assess the extent of genotoxicity [22,35]. This assay evaluates the damage through DNA double-stranded breaks, resulting in faster electrophoretic migration of denatured and cleaved DNA fragments, yielding a tail-shaped comet. The present study used a comet assay to evaluate the extent of DNA damage (combination of single-strand breaks, double-strand breaks, and alkaline-labile sites) induced by CMBNPs. The comet score, an average of all the comets from each group, is enlisted in Table 1. Figure 9 shows the visualization of DNA damage by fluorescence microscopy performed with 115 μg/mL CMBNPs and untreated control. CMBNPs were found to increase the comet tail length from 13.63 µm to 45 µm. The comet assay data reaffirms the CMBNPs-mediated cytotoxicity observed by NRU assay. Several studies also reported increased tail length in response to CuO NPs, AuO NPs, and MnO in different cancer cell lines [22,36,37].

A comparison Table 2 listing various bimetallic NPs and current NPs, showing their variable properties and applications.

## 4. Conclusions

This study aimed to efficiently synthesize a nanoparticle that is cheap and eco-friendly via a green route. The data obtained from various characterization techniques indicate the biosynthesis of an efficient bimetallic nanoparticle. To our knowledge, this is the first study to examine the potential anticancer properties of green bimetallic (Cu-Mn) nanoparticles produced from pumpkin seed extract. The biogenically synthesized CMBNPs of ~50 nm from pumpkin seed extract seems to be promising anticancer agents that can be obtained in a green and environmentally friendly way. The treatment with CMBNPs showed a dose-dependent cytotoxicity, inhibited migration of colon adenocarcinoma cells, and showed significant DNA damage. However, the precise mechanism of action of CMBNPs needs to be uncovered. For further exploitation of this nano-formulation, we recommend that future studies on various in vitro and in vivo models. These studies will contribute to the growing body of knowledge regarding the synthesis of composite mixtures as anticancer agents.

## Figures and Tables

**Table 1:** Comet score depicting DNA damage as a result of CMBNPs treatment in HT-29 cells.

	Control (µm)	Treated (µm)
Comet area	6101.28 ± 814.99	8843.92 ± 569.07
Comet length	70.5 ± 5.97	103.51 ± 4.18
Head length	56.87 ± 6.57	58.51 ± 4.71
Head DNA percentage	61.83 ± 5.18	43.98 ± 5.12
Tail length	13.63 ± 2.47	45 ± 5.18
Tail DNA percentage	38.18 ± 5.18	56.03 ± 5.12
Tail moment	11.97 ± 2.36	38.7 ± 4.93
Olive moment	7.05 ± 1.26	23.31 ± 2.69

**Table 2:** Comparison of earlier reported bimetallic NPs and current biosynthesized bimetallic NPs.

Bimetallic Nanoparticles	Species	Application	Shape/Morphology	Size	References
Ag/Au	Amino acid tryptophan	Antitumor effect/cytotoxicity	Cubic/smaller spherical	50–100 nm	[38]
Ag/Au	Alloy and core–shell	Anti-cancerous prototype	Spherical	25–50 nm	[39]
Ag/Au	Amino acid tryptophan	Tumor growth and prevent metastasis in a mouse model	-	-	[16]
Ag-Cu	Leucas aspera	Anticancer activity against alveolar cancer	Tetragonal, smooth-surfaced spherical structures	20 nm	[40]
Ag/Cu and Cu/Zn	Toddy palm	Antitumor, antioxidant, and antibacterial activity		80 nm, 100 nm	[41]
Ag-Au and Ag-Au	Stigmaphyllon ovatum	In vitro anticancer potential	Triangular	23.5 nm, 78 nm 14.9 nm	[42]
Zno-Ag	Laser ablation	Anticancer activity	Hexagonal	30–130 nm	[18]
Cu-Mn	Pumpkin seeds extract	Anticancer activity	Spherical	50 nm	Present work

## Data Availability

The original contributions presented in the study are included in the article. Further inquiries can be directed to the corresponding authors.
